# Non-Foliar Photosynthesis in Pea (*Pisum sativum* L.) Plants: Beyond the Leaves to Inside the Seeds

**DOI:** 10.3390/plants13202945

**Published:** 2024-10-21

**Authors:** Nataliia Stepanova, Tatiana Zhilkina, Anastasia Kamionskaya, Galina Smolikova

**Affiliations:** 1 Federal State Institution Federal Research Centre “Fundamentals of Biotechnology”, Russian Academy of Sciences, 119071 Moscow, Russia; stepanovanataliia.v@yandex.ru (N.S.); akamio@fbras.ru (A.K.); 2 Department of Plant Physiology and Biochemistry, St. Petersburg State University, 199034 St. Petersburg, Russia

**Keywords:** chlorophyll *a* fluorescence, leaves, non-foliar photosynthesis, Pulse-Amplitude-Modulation (PAM) fluorometry, photosynthesis, *Pisum sativum* L., pods, seeds

## Abstract

In addition to leaves, photosynthesis can occur in other green plant organs, including developing seeds of many crops. While the majority of studies examining photosynthesis are concentrated on the leaf level, the role of other green tissues in the production of total photoassimilates has been largely overlooked. The present work studies the photosynthetic behavior of leaves and non-foliar (pericarps, coats, and cotyledons) organs of pea (*Pisum sativum* L.) plants at the middle stage of seed maturation. The Chl *a* fluorescence transient was examined based on OJIP kinetics using the FluorPen FP 110. A discrepancy was observed between the performance index (PI_ABS_) for foliar and non-foliar plant tissues, with the highest level noted in the leaves. The number of absorbed photons (ABS) and captured energy flow (TRo) per reaction center (RC) were elevated in the non-foliar tissues, which resulted in a faster reduction in Q_A_. Conversely, the energy dissipation flux per RC (DIo/RC and PHI_Do) indicated an increase in the overall dissipation potential of active reaction centers of photosystem II. This phenomenon was attributed to the presence of a higher number of inactive RCs in tissues that had developed under low light intensity. Furthermore, the expression of genes associated with proteins and enzymes that regulate ribulose-1,5-bisphosphate carboxylase/oxygenase (RuBisCo) activity was observed, including chaperonins Cpn60α and Cpn60β, RuBisCO activase, as well as phosphoribulokinase. The expression of these genes was found to differ between foliar and non-foliar tissues, indicating that the activation state of RuBisCO may be modified in response to light intensity. Overall, the present study provides insights into the mechanisms by which non-foliar green tissues of plants adapt to efficient light capture and utilization under low light conditions.

## 1. Introduction

Life on Earth depends on photosynthesis, either directly or indirectly. Photosynthesis is a biological process through which photosynthetic organisms convert light into chemical energy to maintain their activities [1,2]. Given the existence of limited agricultural land and the increasing human population, it is essential to enhance the photosynthetic activity of plants to produce more food. Photosynthesis has been extensively researched, and its mechanisms and pathways are well established. However, most studies have focused on leaves, with little attention given to other green tissues as a target for additional carbon acquisition [3].

Nevertheless, green tissues other than leaves, including stems, roots, ears, flowers, pods, seeds, and fruits, have been shown to contribute to total plant carbon gain through their own photosynthetic processes [4,5,6,7,8,9,10,11,12,13]. Aschan and Pfanz [5] differentiated two main groups of photosynthetically active plant organs based on the accessibility of the atmospheric carbon dioxide. Green leaves, stems, and green sterile flower organs are characterized by net photosynthetic assimilation, utilizing mainly atmospheric CO_2_. In contrast, chlorophyll-containing bark and wood tissues, most fruits, roots, and fertile flower organs are primarily subordinated to non-photosynthetic functions, but typically perform an effective internal CO_2_ recycling during photochemical reactions using both the atmospheric and respirationally released CO_2_. For this reason, non-foliar photosynthesis often manifests as net photosynthesis or refixation of the CO_2_ produced internally by the cellular metabolic processes.

According to Wang et al. [14], under leaf photosynthesis inhibition, the seed yield, number of siliques per plant, and number of seeds per silique can be reduced by about 30%. But while leaves serve as the primary photosynthetic organs in plants, non-foliar tissues can supply up to 60% of their total carbon demand through fixation of their own CO_2_ [5]. Specifically, Cho et al. [7] have shown that soybean pods and seeds can contribute up to 9% of the total daily carbon gain through photosynthesis. 

It has also been demonstrated that the contribution of non-foliar photosynthesis becomes more significant when plants are subjected to stressors [9,12,13,15]. It is therefore crucial to comprehend how plants adapt their photosynthetic processes to environmental cues through the enhancement of non-foliar tissue function in order to facilitate the breeding of stress-tolerant crops.

The pea (*Pisum sativum* L.) is an important crop in the agricultural industry. Since Mendel’s discovery of the laws of inheritance, the pea has continued to be the subject of scientific interest as a source of protein, starch, fiber, and minerals. In this study, we conducted the comparative analysis of the photosynthetic behavior between leaves and non-foliar green plant organs, with a particular focus on leaves, pericarps, coats, and cotyledons. The methodology employed was that of PAM fluorometry, coupled with an analysis of the expression of genes related to ribulose-1,5-bisphosphate carboxylase/oxygenase (RuBisCo) activity. 

The novelty of this work lies in comparing the photochemical activity in plant tissue with different availabilities of light source. Despite extensive research in the field of non-foliar photosynthesis, there is still much to be discovered about the photosynthetic behavior of seed embryos covered with coats and pericarps. Recently, we showed that photochemically active radiation (PAR) reaching the cotyledons at the photochemical active middle stage of seed maturation was characterized by a high proportion of green and far-red light, with a low percentage of red light and no blue light [16]. However, regardless of low light intensity and untypical spectral ranges of PAR, pea embryos are photochemically active [17,18]. Embryonic chloroplasts are responsible for producing NADP(H) and ATP, which are then used to convert sucrose to acetyl-CoA and then further convert them to fatty acids [19,20]. Therefore, the intensity of embryonic photochemical reactions has a significant impact on the efficiency of reserve nutrient accumulation [21]. Understanding the differences in photochemical behavior between foliar and non-foliar plant tissue will help to comprehend how the deep-lying plant organs adapt to utilize light in photochemical reactions.

## 2. Materials and Methods

### 2.1. Plant Material and Growth Conditions

**Figure 1:**
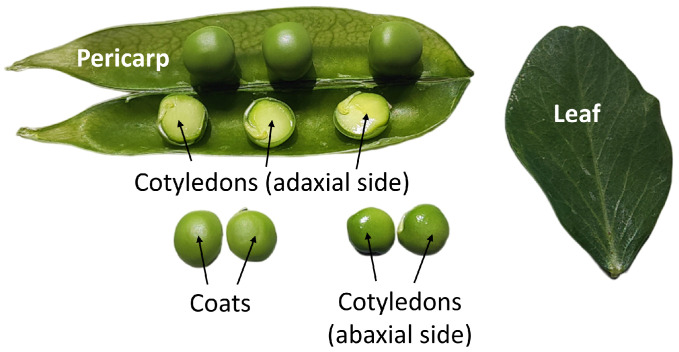
Images of tissues from *P. sativum* plants used for the analysis. Seeds represent the middle stage of maturation.

Pea (*Pisum sativum* L.) plants were grown in an experimental climate control facility at the Institute of Bioengineering (Research Center of Biotechnology, Russian Academy of Sciences). Commercial seeds of the vegetable cv. Gloria were sown in 2 L round pots filled with the All-Mix BioBizz substrate (BioBlitz World-wide Organics, Drachten, The Netherlands). The spectral characteristics of the light were measured using the UPRtek PG100N spectroradiometer (UPRtek, Miaoli County, Taipei City, Taiwan). The photosynthetic photon flux density (PPFD) of illumination was 137 ± 7 µmol photons m^−2^ s^−1^. The photosynthetic photon fluxes in ranges between 400–500 nm (PFD-B), 500–600 nm (PFD-G), and 600–700 nm (PFD-R) were 32 ± 2 µmol photons m^−2^ s^−1^, 35 ± 2 µmol photons m^−2^ s^−1^, and 70 ± 4 µmol photons m^−2^ s^−1^, respectively (Figure S1). Three pods with five plants per pod were cultivated at 24 ± 2 °C and in a 16 h photoperiod under white fluorescent LEDs (Bridgelux, Inc., Moscow, Russia). Plants were grown until the middle stage of seed maturation (Figure 1).

### 2.2. Chlorophyll Content

Relative chlorophyll concentration was determined non-invasively using the LEAF CHL PLUS portable chlorophyll meter (FT Green LLC, Wilmington, DE, USA). The instrument uses an optical measurement system based on the difference in optical density at two wavelengths (640 nm and 940 nm). The data are presented in atLEAF units and then converted to mg/cm^2^ according to the calculations presented on the website https://www.atleaf.com/SPAD, accessed on 15 July 2024. 

### 2.3. Chlorophyll a Fluorescence

A FluorPen FP 110 fluorometer (Photon Systems Instruments, Drasov, Czech Republic) was used to measure the photosynthetic parameters in pea plants. It was equipped with a blue LED emitter (455 nm) and was focused on light intensities of up to 3000 μmol m^−2^ s^−1^. The OJIP protocol was used to capture rapid chlorophyll fluorescence transience, and the NPQ protocol was used to quantify photochemical and non-photochemical quenching of chlorophyll fluorescence. Leaves, pericarps, coats, and cotyledons were isolated from the pea plants at the middle stage of seed maturation and kept in light-proof boxes on moist filter paper for 30 min for dark adaptation. They were later subjected to a 3000 μmol m^−2^ s^−1^ blue light pulse (adjusted to 80%), with the detection of minimum (Fo) and maximum (Fm) values of Chl fluorescence. These values were used to estimate photosystem II (PSII) bioenergetic indices according to the methods described by [22,23].

### 2.4. RNA Isolation and Real-Time PCR Analysis

Leaves, pericarps, coats, and cotyledons of pea plants were harvested from pea plants at the middle stage of seed maturation. The peripheral and interior parts of the cotyledons (abaxial and adaxial regions, relatively) were dissected before analysis. Isolated tissues were frozen in liquid nitrogen, ground in a pre-cooled mortar with a pestle, and stored at −80 °C until required.

Total RNA was extracted from 100 mg of tissue using ExtractRNA reagent according to the Evrogen protocol (https://evrogen.ru/support/technologies/rna-isolation, accessed on 15 July 2024). RNA was used for first-strand cDNA synthesis with an oligo-dT primer and the MMLV RT kit (Evrogen, Moscow, Russia). RNA and cDNA concentrations were determined using fluorometry (Eppendorf BioSpectrometer, Eppendorf AG, Hamburg, Germany). A CFX96 real-time PCR detection system (Bio-Rad Laboratories, Hercules, CA, USA) was used to measure target gene expression. Threshold values (CT) were used to quantify relative gene expression using the comparative 2^−ΔΔCT^ method [24]. Each sample was normalized using the CT value of the protein phosphatase PP2A regulatory subunit A gene (GenBank Z25888) as a reference. All primer pairs (Table S1) were designed using the Vector NTI program and were produced by Evrogen company (Moscow, Russia). 

### 2.5. Statistical Analysis

Statistical analysis was performed using an MS Excel add-in. Data are expressed as mean ± standard deviation (SD). Significance of differences between leaves and non-foliar tissues was estimated using Student’s two-tailed *t*-test (α = 0.05, *p* < 0.05). Ten and seventeen biological replicates were used to calculate relative chlorophyll content and photosynthetic performance parameters, respectively. The averages of gene expression levels in the qPCR analysis were derived from the three replicates.

## 3. Results

### 3.1. Chlorophyll Content and OJIP Chlorophyll Fluorescence Kinetics in Pea Photosynthetic Tissues

To understand the differences in the photochemical activity between the foliar (leaves) and non-foliar (pericarps, coats, cotyledons) tissues of pea plants, we initially analyzed their chlorophyll content and kinetics of chlorophyll fluorescence. Plants exhibiting green pods and seeds at the middle stage of maturation were selected for analysis (Figure 1).

**Figure 2:**
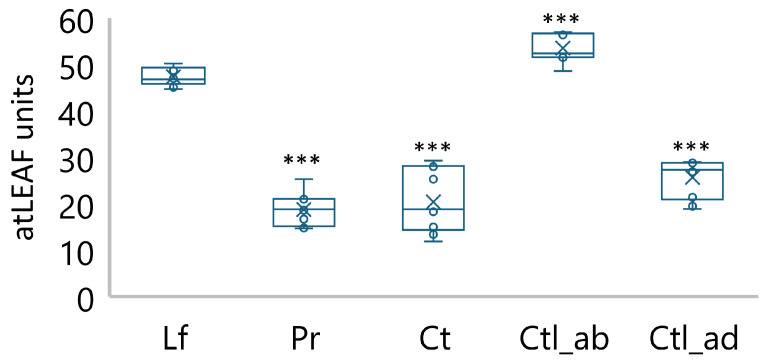
Relative chlorophyll content estimated in pea plants using the portable chlorophyll meter at LEAF CHL PLUS (FT Green LLC, USA): Lf—leaves; Pr—pericarps; Ct—coats; Ctl_ab—abaxial region of cotyledons (periphery); Ctl_ad—adaxial region of cotyledons (inside). Box plots calculated from ten biological replicates. Significant differences compared to leaves are indicated (*** *p* ≤ 0.001).

To measure the chlorophyll content, we used a non-invasive approach with the portable chlorophyll meter atLEAF CHL PLUS (FT Green LLC, Washington, DC, USA). The relative chlorophyll concentration in the leaves was 47.4 atLEAF units (or 0.03 mg/cm^2^) (Figure 2). 

The value of atLEAF units was observed to be 2.5 times lower in the pericarp, 2.3 times lower in the coats, and 1.8 times lower in the adaxial region of the cotyledons. It is noteworthy that the chlorophyll level at the periphery of the cotyledons (abaxial region) was found to be 2.9 times higher than in the pericarps and 2.6 times higher than in the coats, which was an unexpected finding.

**Figure 3:**
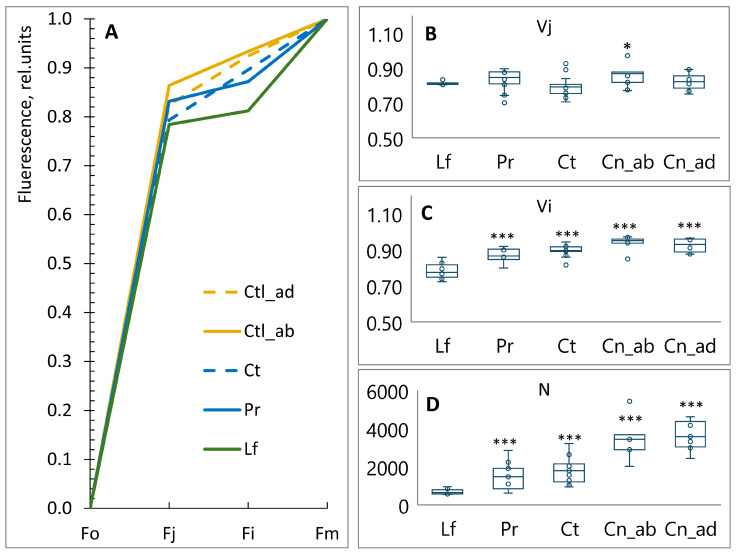
OJIP chlorophyll fluorescence kinetics in leaves (Lf), pericarps (Pr), coats (Ct), abaxial region of cotyledons (Ctl_ab), and adaxial region of cotyledons (Ctl_ad) of pea plants: (**A**) Chl fluorescence curve. Fo is fluorescence intensity at 50 μs. F_J_ is fluorescence intensity at 2 ms. Fi is fluorescence intensity at 30 ms. Fm is maximal measured fluorescence intensity. The data were scaled from zero to one. (**B**) Vj is relative variable fluorescence at the J step. (**C**) Vi is relative variable fluorescence at the I step. (**D**) N is. Box plots calculated from 17 biological replicates. Outliers are represented as white dots. Significant differences compared to leaves are indicated (*** *p* ≤ 0.001, * *p* ≤ 0.05).

The OJIP chlorophyll fluorescence kinetics were quantified using a PAM-fluorometric approach with a FluorPen FP 110 (PSI, Drasov, Czech Republic). Figure 3A demonstrates three distinct plateaus, each corresponding to a specific step in the electron transfer within the electron transport chain of chloroplasts (ETC). Following the application of actinic light, the intensity of the chlorophyll fluorescence increases from the initial value (Fo) to the maximum (Fm) through two intermediate phases (‘J’ and ‘I’). The fluorescence intensity at 50 μs was considered to be Fo, i.e., the intensity when all reaction centers of PSII (PSII RCs) are open. The maximum fluorescence intensity (Fm) is provided, showing that the excitation intensity is high enough to allow for the closure of PSII RCs. The fluorescence intensity at 2 ms is denoted as Fj and, at 30 ms, as Fi. The data were normalized between Fo and Fm to facilitate the most accurate comparisons of the shapes of the curves. 

The chlorophyll fluorescence exhibited a rapid increase from the initial level (Fo) to the Fj. This phase corresponds to the transfer of electrons from the PSII RC to plastoquinone Q_A_. The Fj is achieved following the reduction in Q_A_, and the Fi is achieved after the reduction in Q_B_. The complete reduction in the pool of plastoquinones results in the achievement of Fm. The Vj and Vi represent the fractions of PSII RCs that are closed after a single charge separation at 2 ms and 30 ms, respectively (Figure 3B,C). No significant differences were observed between the studied plant tissues at the stage ‘J’. However, at the stage ‘I’, the amount of closed PSII RCs was higher in the cotyledons, coats, and pericarps than in the leaves. The pool of reduced Q_A_ is expressed as turn-over number (N) (Figure 3D). N indicates the number of times PSII RCs were closed and re-opened between Fo and Fm. Compared to leaves, N was 2.1 and 2.7 times higher in pericarps and coats and 5.3 and 5.8 times higher in abaxial and adaxial regions of the cotyledons, respectively.

### 3.2. Specific Energy Fluxes and Photosynthetic Performance Index

**Figure 4:**
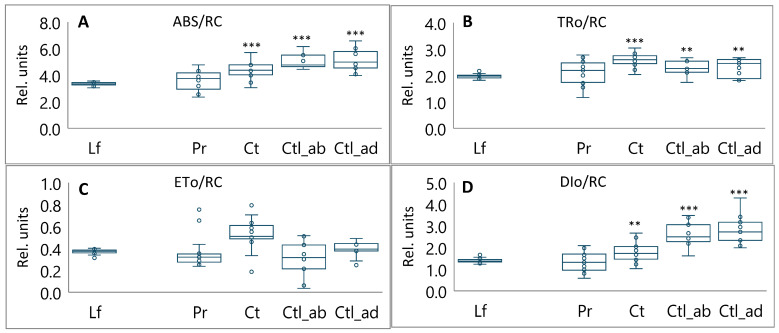
Specific energy fluxes per active reaction center (RC) in leaves (Lf), pericarps (Pr), coats (Ct), abaxial region of cotyledons (Ctl_ab), and adaxial region of cotyledons (Ctl_ad) of pea plants: (**A**) ABS/RC—absorption flux/effective antenna size (rel.units); (**B**) TRo/RC—trapped energy flux leading to a reduction in Q_A_ (rel.units); (**C**) ETo/RC—electron transport flux further than Q_A_ (rel.units); (**D**) DIo/RC—dissipation flux (rel.units). Box plots calculated from 17 biological replicates. Outliers are represented as white dots. Significant differences compared to leaves are indicated (*** *p* ≤ 0.001, ** *p* ≤ 0.01).

It is noteworthy that the specific energy fluxes per reaction centers were higher in non-foliar tissues compared to leaves (Figure 4). The trapping of excitons, which leads to a reduction in Q_A_ (TRo/RC), was increased from 1.2 to 1.3 (Figure 4B). This was accompanied by a proportional elevation of absorption fluxes (ABS/RC) (Figure 4A). ABS is the amount of photon flux absorbed by the pigments in the antenna complex and is, therefore, related to the effective antenna size. Nevertheless, the electron transport flux beyond Q_A_ (ETo/RC) remained relatively unchanged (Figure 4C), with the trapped energy dissipated through non-photochemical means (DIo/RC) (Figure 4D). 

**Figure 5:**
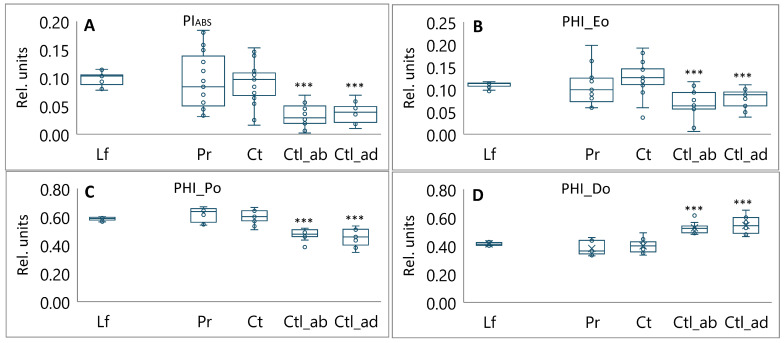
Photosynthetic performance index, yield, and overall flux ratios in leaves (Lf), pericarps (Pr), coats (Ct), abaxial region of cotyledons (Ctl_ab), and adaxial region of cotyledons (Ctl_ad) of pea plants: (**A**) PI_ABS_—performance index on absorption basis; (**B**) PHI_Eo—overall electron transport potential of the active PSII RCs; (**C**) PHI_Po—the maximum quantum yield of primary photochemistry; (**D**) PHI_Do—the potential energy dissipation flux. Box plots calculated from 17 biological replicates. Outliers are represented as white dots. Significant differences compared to leaves are indicated (*** *p* ≤ 0.001).

The probability of a trapped exciton moving an electron into the electron transport chain (PHI_Eo) was significantly reduced in the abaxial and adaxial regions of the cotyledons, with values between 0.6 and 0.7, respectively (Figure 5B). This phenomenon may be responsible for the observed accumulation of Q_A_ (N) (Figure 3D), the low performance index PI_ABS_ (Figure 5A), and the low maximum quantum yield of primary photochemistry (PHI_Po) (Figure 5C). Conversely, the dissipation per active reaction center (DIo/RC) exhibited a twofold increase in cotyledons (Figure 4D). Moreover, the potential energy dissipation flux (PHI_Do) (Figure 5D) exhibited a 1.3-fold increase. Non-photochemical quenching prevented photoinhibition by limiting the amount of excitation energy received by the light-harvesting complexes of PSII.

### 3.3. Expression of Genes Related to RuBisCO Activity

To elucidate the differences in the photosynthetic activity between the leaves and non-foliar (pericarps, coats, cotyledons) organs of pea plants, we analyzed the expression of genes related to ribulose-1,5-bisphosphate carboxylase/oxygenase (RuBisCo) activity. For the purpose of qRT-PCR analysis, the genes encoding RuBisCO large subunit-binding protein subunit alpha (Cpn60α), RuBisCO large subunit-binding protein subunit beta (Cpn60β), RuBisCO activase (RCA), and phosphoribulokinase (PRK) were selected. Total RNA was isolated from the tissues previously used for the PAM-flurometrical study. 

**Figure 6:**
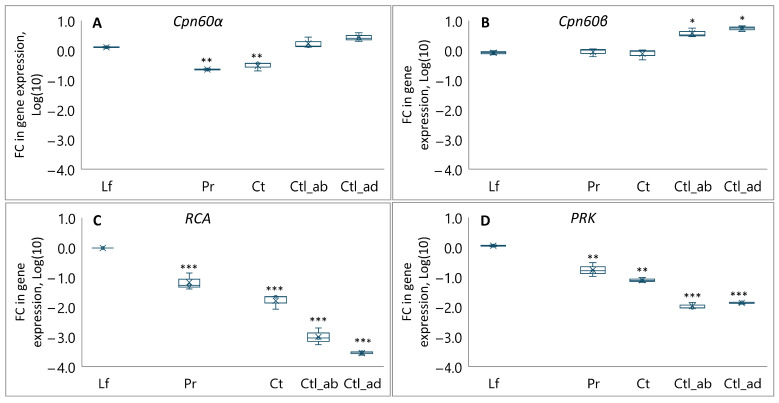
Relative expression of target genes related to RuBisCo structure (A, B) and activity (C, D) in leaves (Lf), pericarps (Pr), coats (Ct), abaxial region of cotyledons (Ctl_ab), and adaxial region of cotyledons (Ctl_ad) of pea plants. (**A**) RuBisCO large subunit-binding protein subunits alpha (Cpn60α). (**B**) RuBisCO large subunit-binding protein subunits beta (Cpn60β). (**C**) RuBisCO activase (RCA). (**D**) Phosphoribulokinase (PRK). Data were normalized to the expression of the protein phosphatase PP2A regulatory subunit A gene (GenBank Z25888) encoding the phosphoprotein phosphatase 2A. Y-axis corresponds to the difference in a gene relative to the mean expression on a log(10)-scale: values above zero-level represent up-regulation, and below it represent down-regulation. Box plots calculated from three biological replicates. Significant differences compared to leaves are indicated (*** *p* ≤ 0.001, ** *p* ≤ 0.01, * *p* ≤ 0.05).

In the course of our experiments, a comparison was conducted between the level of gene expression in the leaves (which served as a control) and that in the pod tissues. The expression of Cpn60α was observed to be sixfold lower in pericarps and fourfold lower in coats, while no notable alterations were observed in the cotyledons (Figure 6A). In contrast, the expression of Cpn60 β was observed to be five and seven times higher in the abaxial and adaxial regions of the cotyledons, respectively, with no significant alterations observed in the pericarps and coats (Figure 6B).

The RCA gene exhibited a notable alteration in its expression level in non-foliar tissues relative to that observed in leaves (Figure 6C). The reduction in light penetration into the tissues was accompanied by a corresponding decline in gene expression, with pericarp tissues showing a 12-fold reduction and cotyledons displaying a 3000-fold reduction.

Similarly to the findings observed for the RCA, the PRK gene also exhibited a notable decrease in expression levels in non-foliar tissues (Figure 6D). The expression was found to be 6-fold lower in pericarps, 14-fold lower in coats, and approximately 100-fold lower in cotyledons.

## 4. Discussion

The pea is a significant legume crop with high nutritional value and a notable capacity for biological nitrogen fixation. Furthermore, it has served as a model for genetic studies since the discovery of Mendel’s laws of inheritance. As all biomass is derived from the photosynthetic activity, the enhancing carbon fixation represents a fundamental objective in the pursuit of increased crop yields. However, the majority of studies have concentrated on leaves, with minimal attention directed toward other green tissues as potential sources for additional carbon acquisition. 

In this study, we analyzed the photosynthetic behavior of the foliar and non-foliar tissues of pea plants, with a particular focus on leaves (which served as a control), pericarps, coats, and cotyledons (abaxial and adaxial regions) (Figure 1). All tissues were observed to be green and contained chlorophylls, as measured non-invasively with a portable chlorophyll meter (Figure 2). The presence of chlorophylls *a* and *b* in the pods, seeds, and embryos of various species has been demonstrated in numerous studies [17,18,25,26,27,28,29]. We have previously shown that the synthesis of chlorophylls in *P. sativum* embryos occurs at the earliest stages of embryogenesis, continues at the middle stages, and stops at late maturation [17,18]. Generally, the total chlorophyll content is lower in embryos than in leaves. This is related with the lower amount of light transmitted to cotyledons through the covering tissues [16]. In our experiments, a comparison of the relative chlorophyll concentration between leaves and non-foliar tissues revealed that the latter exhibited a significantly lower concentration in pericarp, coats, and inside of cotyledons (adaxial region). However, the concentrations in the periphery of cotyledons (abaxial region) was found to be approximately three times higher than that in pericarps and coats (Figure 2). This phenomenon is regarded as a means by which cotyledons sustain considerable photochemical activity in low light intensities.

The process of photosynthesis is subject to rigorous regulation and must be synchronized with the physiological state of the photosynthetic organs [22]. This was evidenced by the discrepancy in the performance index (PI_ABS_) values observed for foliar and non-foliar plant tissues with different functionalities. The highest level of PI_ABS_ was observed in the leaves, followed by a notable decline in non-foliar tissues, with the lowest value observed in the cotyledons (Figure 5). PI_ABS_ is indicative of the functionality of both PS I and II and provides valuable quantitative information regarding the current state of tissue performance. Notably, the TRo/RC value was elevated in the non-foliar tissues, reaching 1.1-fold in the pericarps, 1.3-fold in the coats, and 1.2-fold in the cotyledons (Figure 4). The TRo/RC value represents the captured energy flow per RCs, which ultimately results in a reduction in Q_A_. This indicates that these non-foliar tissues possess functional active RCs capable of primary charge separation.

The plastoquinone (PQ) pool was reduced more rapidly in non-foliar tissues, as indicated by an increase in the Vi value (relative variable fluorescence at the ‘J’ step of OJIP chlorophyll fluorescence kinetics) (Figure 3C). In coats and cotyledons, this phenomenon is related to a lower amount of PQ, a consequence of these tissues developing under lower light intensity. In pericarps, this could be related to the low number of chlorophylls (Figure 2). Furthermore, the increase in N (the number of times the reaction centers of PSII were closed and reopened) indicated that the re-oxidation of Q_A_ in non-foliar tissues was 2–3 times more efficient in pericarps and coats and 5 times more efficient in cotyledons than in leaves (Figure 3D). The electron transport flux from Q_A_ to Q_B_ and beyond (ETo/RC) in non-foliar tissues was found to be identical to that observed in leaves (Figure 4C), indicating that non-foliar tissues are capable of performing multiple turnovers.

A significant variation exists in the number of photons absorbed by the pigment-containing antenna complex (ABS) per RCs, which are subsequently interpreted as the effective antenna size. The ABS/RC ratio was found to be 1.1 times higher in the pericarp, 1.3 times higher in the coats, and 1.5 times higher in the cotyledons compared to leaves (Figure 4A). This suggests that a combination of light-harvesting complexes may be undergoing changes that enable them to function effectively in low light conditions. An increase in the ABS/RC value was found to be correlated with an increase in energy dissipation flux per RC (DIo/RC and PHI_Do) (Figure 4D and Figure 5D), reflecting the overall dissipation potential of active PSII RCs. 

It is therefore questionable why there is a reduction in PI_ABS_ in non-foliar tissues when active RCs are capable of primary charge separation and electron transport, and when these processes are not inhibited. This phenomenon may be attributed to the presence of inactive RCs [22]. In the membrane model for specific fluxes developed by Strasser et al. [22], the ABS/RC value represents the total absorption of PSII antenna chlorophylls divided by the number of active RCs (in the sense of Q_A_ reduction). The authors demonstrated that heating leaves to temperatures up to 44 °C resulted in increased ABS/RC (due to the inactivation of some RCs), DIo/RC (due to the high dissipation from inactive RCs), and electron transport per active RC (due to thermal activation of the dark reactions). It is noteworthy that a similar phenomenon was observed in non-foliar tissues, attributed to elevated levels of inactive RCs resulting from low light conditions.

The light reactions facilitate the conversion of energy from sunlight into chemical energy in the form of ATP and NADPH. These are then utilized in the light-independent Calvin–Benson–Bassham cycle to produce carbon compounds and to regenerate the 5-carbon sugar substrate of Rubisco, RuBP [30]. The efficiency of ATP and NADPH utilization in the dark reactions of photosynthesis affects the efficiency of electron transport in thylakoid membranes. It has been established that non-foliar tissues, including seed embryos, contain RuBisCO, which enhances their carbon efficiency [19,21,31,32]. 

We studied the expression of genes associated with proteins and enzymes that regulate RuBisCo activity, including chaperonins Cpn60α, Cpn60β, RuBisCO activase, and phosphoribulokinase, that regenerate the Rubisco substrate (RuBP). RuBisCo from higher plants has been classified as a green-type of form I. It is a hexadecameric complex consisting of eight large (RbcL, ~50–55 kDa) and eight small (RbcS, ~12–18 kDa) subunits [30,33]. RbcL are synthesized within the chloroplast, whereas RbcS are imported across the chloroplast envelope subsequent to synthesis in the cytoplasm. It has been shown that the protein assembly of hexadecameric RuBisCo is mediated by specific assembly chaperones [34,35]. The chloroplast chaperonins (Cpn60) were originally named RuBisCo large subunit binding proteins based on the finding that newly synthesized RbcL subunits interact with a large protein complex. The plastid chaperonins in plants (including *P. sativum*) are typically encoded in the nucleus and consist of two different isoforms, Cpn60α and Cpn60β [23]. It was hypothesized [30] that Cpn60α evolved to specifically recognize and perhaps prioritize RbcL binding in the chloroplast, while Cpn60β retained responsibility for the oligomerization of, and productive interaction with, other chaperonins. In our research, the *Cpn60α* exhibited higher expression in leaves and cotyledons, while *Cpn60β* expression was higher only in cotyledons (Figure 6A,B).

Once assembled, RbcL should undergo carboxylation followed by Mg^2^⁺ binding (carbamylation) [30]. This modification is essential for binding the sugar substrate ribulose-1,5-bisphosphate (RuBP). However, an excess of attached sugar phosphates can impede the enzymatic activity of RuBisCo. This step is regulated by RCA, which facilitates the dissociation of inhibitory sugars in an ATP-dependent manner [34]. 

The rate of photosynthetic carbon fixation is constrained not only by the catalytic efficiency of Rubisco, but also by the capacity for regeneration of its substrate, ribulose-1,5-bisphosphate (RuBP) [36]. Phosphoribulokinase (PRK) is a crucial enzyme in the carbon cycle of photosynthesis, facilitating the ATP-dependent phosphorylation of ribulose-5-phosphate (Ru5P) to RuBP [37]. In light conditions, PRK is regulated through the oxidoreduction in specific disulfides by thioredoxins. But in the dark, PRK forms an inhibitory complex with CP12 protein and glyceraldehyde-3-phosphate dehydrogenase (GAPDH) [38]. 

According to our results, RCA and PRK demonstrated a gradual decline in expression as light intensity decreased. The activity of both enzymes is dependent on ATP synthesized during light reactions. RCA utilizes energy derived from ATP hydrolysis to promote a conformational alteration in RuBisCo, thereby triggering the opening of its catalytic sites and releasing inhibitory sugar-phosphate derivatives [34]. PRK facilitates the ATP-dependent phosphorylation of Ru5P to RuBP [37]. As demonstrated by Gurrieri et al. [38], light-dependent regulation of PRK is mediated by redox reactions involving specific disulfides by thioredoxins. Conversely, in the absence of light, PRK forms an inhibitory complex with the CP12 protein and glyceraldehyde-3-phosphate dehydrogenase, preventing its activity. Consequently, the activation state of RuBisCO can be modified in response to light intensity, thereby regulating crop response to environmental cues.

Aschan and Pfanz [5] distinguished between photosynthetically active plant organs, categorizing them into two groups based on the accessibility of atmospheric carbon dioxide. The findings of this study allow us to categorize photosynthetically active plant organs into three groups based on their primary functions and light availability. The first group comprises leaves, which are primarily responsible to producing organic substances through photosynthesis. They exhibit a high concentration of photosynthetic pigments and chloroplasts with well-developed thylakoid membranes, utilize atmospheric CO_2_ as a carbon source, and engage in photochemical reactions under high light intensity [2]. The second group comprises non-foliar green tissues located on the periphery of various plant organs (stems, pods, fruits) [9,39]. In this case, these include pericarps and coats of pods. Compared to leaves, they exhibit a lower level of chlorophylls, but have easy access to both atmospheric CO_2_ and sunlight. While their primary function is not photosynthesis, they can provide organic substances to the embryos inside the pods through their own photosynthetic processes. The third group comprises the deep-located tissues, particularly the chlorenchymal tissues of the inner cortex [10,40] and embryos of chlorophyll-containing seeds [41,42,43]. These tissues utilize sucrose supplied from the mother plant and CO_2_ released through respiration as a carbon source [19,44] and engage in photochemical reactions under low irradiance and spectral ranges untypical of leaf photosynthesis [16]. In order to adapt to a low amount of light, they develop plastids with well-formed thylakoid systems, primarily located in the subepidermal cells [18]. 

Thus, despite the fact that the primary function of non-foliar tissues is not photosynthesis, they can perform photochemical reactions even under low light conditions. This enables plants to produce more ATP and NADPH, thereby supplying themselves with additional energy. It is noteworthy that, compared to leaves, cotyledons exhibit a higher level of chlorophyll at the periphery, possess an increased ABS/RC value related to the ‘antenna size’, an increased ratio of inactive RCs, and a high energy dissipation flux per active RCs. This adaptation is likely driven by the need for efficient light capture and utilization.

## Data Availability

Data are contained within the article and Supplementary Materials.

## Supplementary Materials

The following supporting information can be downloaded at: https://www.mdpi.com/article/10.3390/plants13202945/s1, Figure S1: Close-type installation for growing the *P. sativum* plants; Table S1: Primers used in this work.
