# Supplementary material for: Non-Foliar Photosynthesis in Pea (Pisum sativum L.) Plants: Beyond the Leaves to Inside the Seeds
Source: Plants (Basel). 2024 Oct 21;13(20):2945. doi: 10.3390/plants13202945 (PMC11511552; doi:10.3390/plants13202945)
Supplement: Supplementary file 1 [file plants-13-02945-s001.zip › plants-3235710-supplementary.pdf]

plants

Supplementary Figures and Tables

Non-Foliar Photosynthesis in Pea (*Pisum sativum* L.) Plants: Beyond the Leaves to Inside the Seeds

Nataliia Stepanova, Tatiana Zhilkina, Anastasia Kamionskaya and Galina Smolikova*

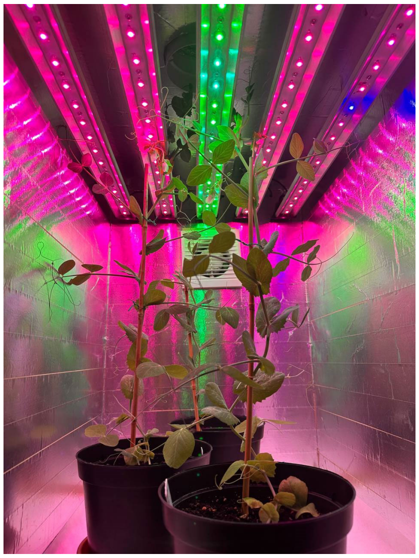

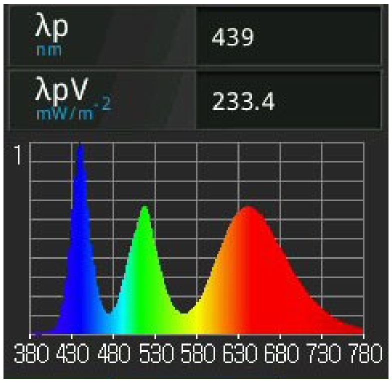

Spectral characteristics of the light measured using the spectroradiometer UPRtek PG100N (Taiwan, China)

Figure S1. Close-type installation for growing the *Pisum sativum* L. plants illuminated with blue LEDs (BL, 400-500 nm, maxima of 450 nm, 32 ± 2 µmol photons m^{-2} s^{-1}), red LEDs (RL, 600-700 nm, maxima of 660 nm, 70 ± 4 µmol photons m^{-2} s^{-1}) and green LEDs (GL, 500-600 nm, maxima of 520 nm, 35 ± 2 µmol photons m^{-2} s^{-1}).

Table S1. List of primers used to analyze the expression of genes related to RuBisCO

Gene symbol (NCBI)	Gene description	Forward(5'-3')	Reverse(5'-3')
LOC127107501	RuBisCO large subunit-binding protein subunit alpha, chloroplastic [*Pisum sativum* (garden pea], 60 kDa chaperonin subunit alpha, CPN-60 alpha	AATGTCGCTGCCAT CAAAGC	TGGAACTCAGCAC-CTGTCAA
LOC127119762	RuBisCO large subunit-binding protein subunit beta, chloroplastic [*Pisum sativum* (garden pea], 60 kDa chaperonin subunit beta, CPN-60 beta	TGCCATCCTAACAG-GAGGTACT	ATATTCTT-GCTCTGCGGCCT
LOC127083843	RuBisCO activase, ribulose bisphosphate carboxylase/oxygenase activase, chloroplastic-like [*Pisum sativum* (garden pea)]	CCAAGATGG-GAATCAACCCCATT A	ATGTCAGCAGCTTCAC-GGTA
LOC12711981	Phosphoribulokinase, chloroplastic [*Pisum sativum* (garden pea)]	CTGGTCTTTT-GGATGCTCCTGA	ACGTTCTGCCATGT-CTCTCTGA
